# 4-Hex­yloxy-3-methoxy­benzaldehyde

**DOI:** 10.1107/S1600536809017358

**Published:** 2009-05-14

**Authors:** Asghar Abbas, M. Khawar Rauf, Michael Bolte, Aurangzeb Hasan

**Affiliations:** a Department of Chemistry, Quaid-i-Azam University Islamabad, 45320-Pakistan; b Institut für Anorganische Chemie, J. W. Goethe-Universität Frankfurt, Max-von-Laue-Str. 7, 60438 Frankfurt/Main, Germany

## Abstract

The title compound, C_14_H_20_O_3_, is a synthetic analogue  with a long aliphatic side chain of the important food additive and flavoring agent, vanillin. There are two independent mol­ecules in the asymmetric unit, each having an essentially planar conformation (r.m.s. deviations of 0.023 and 0.051Å for all non–H atoms of the two mol­ecules in the asymmetric unit).

## Related literature

Schiff-base derivatives (Guo *et al.*, 2008 ▶), metal complexes (Neelakantan *et al.*, 2008 ▶) and 2-amino-4-phenylthiazole derivatives (Ashalekshmi *et al.*, 2008 ▶) of vanillin have shown potential antibacterial activity. Bromovanin (6-bromine-5-hydroxy-4-methoxybenzaldehyde) (Yan *et al.*, 2007 ▶) and caffeate analogues (Xia *et al.*, 2008 ▶) derived from vanillin exhibit a potent anti-proliferative effect on a broad spectrum of cancer cell lines. For the biological activity of vanillin, see: Liang *et al.* (2009 ▶), and for glycosides of vanillin, see: Charles *et al.* (2009 ▶); Lim *et al.* (2008 ▶). For details of the synthesis, see: Williamson (1852 ▶). For related structures, see: Li (2008 ▶). For bond-length data, see: Allen *et al.* (1987 ▶).
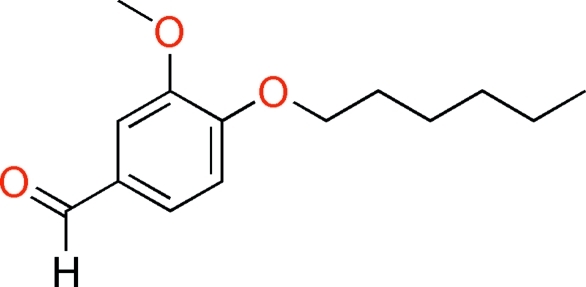


## Experimental

### 

#### Crystal data

C_14_H_20_O_3_
*M* *_r_* = 236.30
Triclinic, 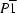

*a* = 9.2788 (5) Å
*b* = 9.3894 (6) Å
*c* = 15.8501 (9) Å
α = 88.099 (5)°
β = 75.065 (5)°
γ = 80.262 (5)°
*V* = 1314.95 (13) Å^3^
*Z* = 4
Mo *K*α radiation
μ = 0.08 mm^−1^
*T* = 173 K
0.42 × 0.37 × 0.36 mm

#### Data collection

STOE IPDS II two-circle diffractometer
Absorption correction: none
17943 measured reflections
4919 independent reflections
3862 reflections with *I* > 2σ(*I*)
*R*_int_ = 0.046

#### Refinement

*R*[*F* ^2^ > 2σ(*F* ^2^)] = 0.049
*wR*(*F* ^2^) = 0.144
*S* = 1.06
4919 reflections
309 parameters
H-atom parameters constrained
Δρ_max_ = 0.69 e Å^−3^
Δρ_min_ = −0.28 e Å^−3^

### 

Data collection: *X-AREA* (Stoe & Cie, 2001 ▶); cell refinement: *X-AREA*; data reduction: *X-AREA*; program(s) used to solve structure: *SHELXS97* (Sheldrick, 2008 ▶); program(s) used to refine structure: *SHELXL97* (Sheldrick, 2008 ▶); molecular graphics: *XP* in *SHELXTL-Plus* (Sheldrick, 2008 ▶); software used to prepare material for publication: *SHELXL97*.

## Supplementary Material

Crystal structure: contains datablocks I, global_rauf53. DOI: 10.1107/S1600536809017358/hg2506sup1.cif

Structure factors: contains datablocks I. DOI: 10.1107/S1600536809017358/hg2506Isup2.hkl

Additional supplementary materials:  crystallographic information; 3D view; checkCIF report

## Figures and Tables

**Fig. 1.**
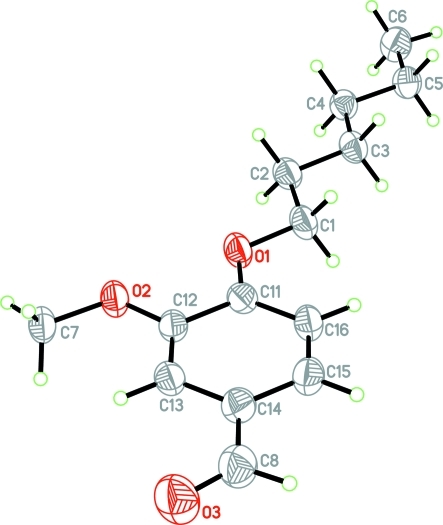
Molecular structure of (I). Displacement ellipsoids are drawn at the 50% probability level.

## supplementary crystallographic information

### Comment

Vanillin, a well known flavoring agent, is the principal flavor and aroma compound in vanilla. Vanillin and its derivatives have been used as flavoring food additives and precursors for the synthesis of organic compounds, and have been reported to show diverse biological applications. Schiff-base derivatives (Guo *et al.*, 2008), metal complexes (Neelakantan *et al.*, 2008) and 2-amino-4-phenylthiazole derivatives (Ashalekshmi *et al.*, 2008) of vanillin have shown potential antibacterial activities against *E. Coli*, *S. Aureus*, *B. Subtilis*, *P. Aeruginosa*, *K. Pneumoniae*, *B. Megaterium*, *V. Cholerae*, and *S. Typhi*,. Bromovanin (6-bromine-5-hydroxy-4-methoxybenzaldehyde) (Yan *et al.*, 2007) and caffeate analogues (Xia *et al.*, 2008) derived from vanillin exhibits a potent anti-proliferative effect on a broad spectrum of cancer cell lines. Vanillin (Liang *et al.*, 2009) and glycosides of vanillin (Charles *et al.*, 2009), exhibit enzyme inhibition, antioxidant, anti-angiogenic, anti-inflammatory and anti-nociceptive activities (Lim *et al.*, 2008). As part of interest in vanillin derivatives, we now report the crystal structure of the title compound (I). A view of compound (I), is shown in Fig 1. The geometrical parameters for (I) are normal (Allen *et al.*,1987) and consistent with those of recently reported ethyl vanillin structure (Li, 2008).The asymmetric unit consist two conformers, each having almost planar conformation. C8, O1 and O2 deviate from the mean plane (C11–C16) by 0.028 (3), 0.020 (2), and 0.020 (2) A°, respectively. The deviations of C8A, O1A and O2A from the mean plane (C11A–C16A) are 0.001 (3), 0.025 (2) and 0.011 (2) A°, respectively.

### Experimental

Vanillin (4-hydroxy-3-methoxybenzaldehyde) (1.52 g) was dissolved in butan-2-one (20 ml), then added K_2_CO_3_ (1.38 g), heated at 60°C and stirred for half an hour. 1-Bromohexane (1.65 g) was added to the reaction mixture and refluxed for 3–4 h on an oil bath (Williamson, 1852). The progress of the reaction was monitored by TLC. Once the reaction was completed, the product was extracted in diethyl ether, solvent evaporated under reduced pressure and crystallized from dichloromethane to get the title compound (I).

### Refinement

Hydrogen atoms bonded to C were included in calculated positions and refined as riding on their parent C atom with C_aromatic_—H = 0.95 Å, C_methylene_—H = 0.99 Å, *U*_iso_(H) = 1.2U(C_eq_) or C_methyl_—H = 0.98 Å. The highest peak in the final difference density map (0.68 e Å^-3^) is located at 0.76Å from H8A.

### Crystal data

**Table:**

C_14_H_20_O_3_	*Z* = 4
*M**_r_* = 236.30	*F*(000) = 512
Triclinic, *P*1	*D*_x_ = 1.194 Mg m^−^^3^
Hall symbol: -P 1	Mo *K*α radiation, λ = 0.71073 Å
*a* = 9.2788 (5) Å	Cell parameters from 16295 reflections
*b* = 9.3894 (6) Å	θ = 3.5–25.9°
*c* = 15.8501 (9) Å	µ = 0.08 mm^−^^1^
α = 88.099 (5)°	*T* = 173 K
β = 75.065 (5)°	Block, colourless
γ = 80.262 (5)°	0.42 × 0.37 × 0.36 mm
*V* = 1314.95 (13) Å^3^	

### Data collection

**Table:**

STOE IPDS II two-circle-diffractometer	3862 reflections with *I* > 2σ(*I*)
Radiation source: fine-focus sealed tube	*R*_int_ = 0.046
graphite	θ_max_ = 25.6°, θ_min_ = 3.4°
ω scans	*h* = −11→11
17943 measured reflections	*k* = −11→11
4919 independent reflections	*l* = −19→19

### Refinement

**Table:**

Refinement on *F*^2^	Primary atom site location: structure-invariant direct methods
Least-squares matrix: full	Secondary atom site location: difference Fourier map
*R*[*F*^2^ > 2σ(*F*^2^)] = 0.049	Hydrogen site location: inferred from neighbouring sites
*wR*(*F*^2^) = 0.144	H-atom parameters constrained
*S* = 1.06	*w* = 1/[σ^2^(*F*_o_^2^) + (0.0794*P*)^2^ + 0.213*P*] where *P* = (*F*_o_^2^ + 2*F*_c_^2^)/3
4919 reflections	(Δ/σ)_max_ = 0.001
309 parameters	Δρ_max_ = 0.69 e Å^−^^3^
0 restraints	Δρ_min_ = −0.28 e Å^−^^3^

### Special details

**Table:**

Geometry. All e.s.d.'s (except the e.s.d. in the dihedral angle between two l.s. planes) are estimated using the full covariance matrix. The cell e.s.d.'s are taken into account individually in the estimation of e.s.d.'s in distances, angles and torsion angles; correlations between e.s.d.'s in cell parameters are only used when they are defined by crystal symmetry. An approximate (isotropic) treatment of cell e.s.d.'s is used for estimating e.s.d.'s involving l.s. planes.
Refinement. Refinement of *F*^2^ against ALL reflections. The weighted *R*-factor *wR* and goodness of fit *S* are based on *F*^2^, conventional *R*-factors *R* are based on *F*, with *F* set to zero for negative *F*^2^. The threshold expression of *F*^2^ > σ(*F*^2^) is used only for calculating *R*-factors(gt) *etc*. and is not relevant to the choice of reflections for refinement. *R*-factors based on *F*^2^ are statistically about twice as large as those based on *F*, and *R*- factors based on ALL data will be even larger.

### Fractional atomic coordinates and isotropic or equivalent isotropic displacement parameters (Å^2^)

**Table:**

	*x*	*y*	*z*	*U*_iso_*/*U*_eq_	
O1	0.65027 (12)	0.60519 (12)	0.99368 (6)	0.0403 (3)	
O2	0.69937 (12)	0.56660 (11)	1.14584 (6)	0.0397 (3)	
O3	1.13475 (16)	0.12211 (15)	1.10444 (9)	0.0648 (4)	
C1	0.62080 (18)	0.63144 (18)	0.90887 (9)	0.0412 (4)	
H1A	0.5870	0.5463	0.8897	0.049*	
H1B	0.7146	0.6475	0.8656	0.049*	
C2	0.50012 (17)	0.76247 (17)	0.91409 (9)	0.0395 (4)	
H2A	0.5342	0.8479	0.9327	0.047*	
H2B	0.4064	0.7468	0.9576	0.047*	
C3	0.46892 (16)	0.78865 (17)	0.82439 (9)	0.0376 (3)	
H3A	0.5625	0.8087	0.7825	0.045*	
H3B	0.4442	0.6990	0.8046	0.045*	
C4	0.34118 (16)	0.91241 (17)	0.82143 (9)	0.0372 (3)	
H4A	0.2474	0.8935	0.8636	0.045*	
H4B	0.3664	1.0029	0.8398	0.045*	
C5	0.31230 (17)	0.93292 (17)	0.73137 (10)	0.0399 (4)	
H5A	0.2874	0.8423	0.7129	0.048*	
H5B	0.4059	0.9522	0.6892	0.048*	
C6	0.1844 (2)	1.05620 (19)	0.72871 (12)	0.0510 (4)	
H6A	0.1701	1.0643	0.6695	0.076*	
H6B	0.2097	1.1468	0.7452	0.076*	
H6C	0.0910	1.0371	0.7697	0.076*	
C7	0.72298 (18)	0.55017 (17)	1.23152 (9)	0.0405 (4)	
H7A	0.7067	0.4537	1.2532	0.061*	
H7B	0.6517	0.6235	1.2707	0.061*	
H7C	0.8268	0.5621	1.2294	0.061*	
C8	1.0993 (2)	0.1514 (2)	1.03692 (11)	0.0514 (4)	
H8	1.1534	0.0935	0.9871	0.062*	
C11	0.75896 (17)	0.49258 (16)	0.99937 (9)	0.0354 (3)	
C12	0.78770 (16)	0.47099 (16)	1.08300 (9)	0.0338 (3)	
C13	0.89766 (16)	0.36073 (16)	1.09506 (9)	0.0359 (3)	
H13	0.9177	0.3470	1.1509	0.043*	
C14	0.98097 (17)	0.26779 (17)	1.02520 (10)	0.0393 (3)	
C15	0.95082 (18)	0.28820 (18)	0.94401 (10)	0.0433 (4)	
H15	1.0063	0.2250	0.8969	0.052*	
C16	0.84110 (18)	0.39934 (17)	0.93083 (9)	0.0403 (4)	
H16	0.8216	0.4122	0.8749	0.048*	
O1A	0.54463 (11)	0.20255 (12)	0.59371 (6)	0.0393 (3)	
O2A	0.57832 (11)	0.17700 (11)	0.75033 (6)	0.0396 (3)	
O3A	1.10136 (16)	−0.29636 (16)	0.61496 (10)	0.0718 (4)	
C1A	0.52746 (17)	0.22746 (18)	0.50646 (9)	0.0390 (3)	
H1A1	0.5165	0.1365	0.4807	0.047*	
H1A2	0.6177	0.2621	0.4693	0.047*	
C2A	0.38839 (16)	0.33951 (18)	0.51114 (9)	0.0384 (3)	
H2A1	0.3969	0.4274	0.5411	0.046*	
H2A2	0.2980	0.3017	0.5456	0.046*	
C3A	0.36975 (16)	0.37813 (18)	0.41991 (9)	0.0382 (3)	
H3A1	0.4591	0.4187	0.3866	0.046*	
H3A2	0.3666	0.2888	0.3894	0.046*	
C4A	0.22797 (17)	0.48624 (18)	0.42019 (9)	0.0395 (3)	
H4A1	0.2298	0.5745	0.4521	0.047*	
H4A2	0.1385	0.4446	0.4522	0.047*	
C5A	0.21109 (17)	0.52794 (18)	0.32935 (10)	0.0415 (4)	
H5A1	0.3006	0.5693	0.2970	0.050*	
H5A2	0.2079	0.4401	0.2975	0.050*	
C6A	0.06916 (19)	0.6370 (2)	0.33156 (11)	0.0489 (4)	
H6A1	0.0631	0.6603	0.2717	0.073*	
H6A2	0.0728	0.7251	0.3618	0.073*	
H6A3	−0.0200	0.5959	0.3626	0.073*	
C7A	0.60438 (19)	0.17584 (18)	0.83582 (10)	0.0449 (4)	
H7A1	0.5974	0.0800	0.8614	0.067*	
H7A2	0.5281	0.2484	0.8730	0.067*	
H7A3	0.7054	0.1984	0.8314	0.067*	
C8A	1.00578 (19)	−0.2192 (2)	0.66439 (14)	0.0553 (5)	
H8A	1.0044	−0.2273	0.7245	0.066*	
C11A	0.65958 (15)	0.09930 (16)	0.60415 (10)	0.0349 (3)	
C12A	0.67768 (15)	0.08413 (16)	0.68992 (9)	0.0337 (3)	
C13A	0.78979 (16)	−0.01963 (16)	0.70729 (10)	0.0380 (3)	
H13A	0.8014	−0.0300	0.7651	0.046*	
C14A	0.88682 (16)	−0.11002 (17)	0.64056 (11)	0.0417 (4)	
C15A	0.87018 (17)	−0.09402 (18)	0.55633 (11)	0.0464 (4)	
H15A	0.9365	−0.1546	0.5108	0.056*	
C16A	0.75726 (17)	0.00993 (18)	0.53781 (10)	0.0427 (4)	
H16A	0.7466	0.0201	0.4798	0.051*	

### Atomic displacement parameters (Å^2^)

**Table:**

	*U*^11^	*U*^22^	*U*^33^	*U*^12^	*U*^13^	*U*^23^
O1	0.0492 (6)	0.0449 (6)	0.0270 (5)	−0.0025 (5)	−0.0137 (4)	0.0013 (4)
O2	0.0483 (6)	0.0419 (6)	0.0271 (5)	0.0031 (4)	−0.0126 (4)	−0.0041 (4)
O3	0.0685 (8)	0.0662 (9)	0.0524 (8)	0.0149 (7)	−0.0189 (6)	0.0002 (6)
C1	0.0522 (9)	0.0488 (9)	0.0261 (7)	−0.0103 (7)	−0.0153 (6)	0.0041 (6)
C2	0.0456 (8)	0.0448 (9)	0.0309 (8)	−0.0121 (7)	−0.0124 (6)	0.0037 (6)
C3	0.0382 (7)	0.0468 (9)	0.0300 (7)	−0.0104 (6)	−0.0105 (6)	0.0027 (6)
C4	0.0374 (7)	0.0426 (9)	0.0329 (8)	−0.0102 (6)	−0.0090 (6)	−0.0014 (6)
C5	0.0394 (7)	0.0451 (9)	0.0368 (8)	−0.0049 (6)	−0.0136 (6)	−0.0009 (6)
C6	0.0560 (10)	0.0499 (10)	0.0481 (10)	0.0034 (8)	−0.0225 (8)	−0.0031 (8)
C7	0.0520 (9)	0.0439 (9)	0.0267 (7)	−0.0031 (7)	−0.0143 (6)	−0.0043 (6)
C8	0.0549 (10)	0.0503 (10)	0.0432 (10)	0.0020 (8)	−0.0084 (8)	−0.0048 (8)
C11	0.0412 (7)	0.0365 (8)	0.0304 (7)	−0.0113 (6)	−0.0098 (6)	0.0025 (6)
C12	0.0391 (7)	0.0359 (8)	0.0267 (7)	−0.0101 (6)	−0.0066 (6)	−0.0015 (6)
C13	0.0419 (8)	0.0390 (8)	0.0286 (7)	−0.0097 (6)	−0.0106 (6)	0.0014 (6)
C14	0.0420 (8)	0.0380 (8)	0.0359 (8)	−0.0077 (6)	−0.0059 (6)	−0.0017 (6)
C15	0.0510 (9)	0.0437 (9)	0.0324 (8)	−0.0092 (7)	−0.0039 (7)	−0.0066 (6)
C16	0.0505 (8)	0.0446 (9)	0.0269 (7)	−0.0106 (7)	−0.0098 (6)	−0.0014 (6)
O1A	0.0377 (5)	0.0495 (6)	0.0293 (5)	0.0023 (4)	−0.0124 (4)	0.0026 (4)
O2A	0.0441 (6)	0.0423 (6)	0.0325 (6)	0.0022 (4)	−0.0157 (4)	−0.0003 (4)
O3A	0.0581 (8)	0.0668 (9)	0.0841 (10)	0.0078 (7)	−0.0173 (7)	−0.0103 (8)
C1A	0.0412 (8)	0.0509 (9)	0.0262 (7)	−0.0060 (7)	−0.0122 (6)	0.0022 (6)
C2A	0.0375 (7)	0.0477 (9)	0.0302 (8)	−0.0037 (6)	−0.0116 (6)	0.0028 (6)
C3A	0.0359 (7)	0.0501 (9)	0.0287 (7)	−0.0047 (6)	−0.0098 (6)	0.0024 (6)
C4A	0.0412 (8)	0.0467 (9)	0.0291 (7)	−0.0010 (6)	−0.0102 (6)	0.0003 (6)
C5A	0.0403 (8)	0.0504 (9)	0.0325 (8)	−0.0037 (7)	−0.0100 (6)	0.0048 (6)
C6A	0.0484 (9)	0.0589 (11)	0.0367 (8)	0.0034 (8)	−0.0142 (7)	0.0039 (7)
C7A	0.0551 (9)	0.0480 (9)	0.0362 (8)	−0.0050 (7)	−0.0220 (7)	0.0008 (7)
C8A	0.0390 (8)	0.0485 (10)	0.0733 (12)	−0.0050 (7)	−0.0055 (8)	−0.0072 (9)
C11A	0.0311 (7)	0.0372 (8)	0.0374 (8)	−0.0056 (6)	−0.0112 (6)	0.0030 (6)
C12A	0.0317 (7)	0.0350 (8)	0.0367 (8)	−0.0081 (6)	−0.0113 (6)	0.0025 (6)
C13A	0.0347 (7)	0.0387 (8)	0.0450 (8)	−0.0091 (6)	−0.0167 (6)	0.0069 (6)
C14A	0.0330 (7)	0.0382 (8)	0.0558 (10)	−0.0081 (6)	−0.0135 (7)	0.0032 (7)
C15A	0.0355 (8)	0.0445 (9)	0.0542 (10)	−0.0032 (7)	−0.0038 (7)	−0.0076 (7)
C16A	0.0385 (8)	0.0496 (9)	0.0386 (8)	−0.0042 (7)	−0.0089 (6)	−0.0030 (7)

### Geometric parameters (Å, °)

**Table:**

O1—C11	1.3502 (18)	O1A—C11A	1.3548 (17)
O1—C1	1.4447 (17)	O1A—C1A	1.4395 (16)
O2—C12	1.3636 (17)	O2A—C12A	1.3622 (17)
O2—C7	1.4298 (16)	O2A—C7A	1.4358 (17)
O3—C8	1.209 (2)	O3A—C8A	1.178 (2)
C1—C2	1.506 (2)	C1A—C2A	1.508 (2)
C1—H1A	0.9900	C1A—H1A1	0.9900
C1—H1B	0.9900	C1A—H1A2	0.9900
C2—C3	1.5266 (19)	C2A—C3A	1.5249 (19)
C2—H2A	0.9900	C2A—H2A1	0.9900
C2—H2B	0.9900	C2A—H2A2	0.9900
C3—C4	1.522 (2)	C3A—C4A	1.519 (2)
C3—H3A	0.9900	C3A—H3A1	0.9900
C3—H3B	0.9900	C3A—H3A2	0.9900
C4—C5	1.519 (2)	C4A—C5A	1.520 (2)
C4—H4A	0.9900	C4A—H4A1	0.9900
C4—H4B	0.9900	C4A—H4A2	0.9900
C5—C6	1.519 (2)	C5A—C6A	1.519 (2)
C5—H5A	0.9900	C5A—H5A1	0.9900
C5—H5B	0.9900	C5A—H5A2	0.9900
C6—H6A	0.9800	C6A—H6A1	0.9800
C6—H6B	0.9800	C6A—H6A2	0.9800
C6—H6C	0.9800	C6A—H6A3	0.9800
C7—H7A	0.9800	C7A—H7A1	0.9800
C7—H7B	0.9800	C7A—H7A2	0.9800
C7—H7C	0.9800	C7A—H7A3	0.9800
C8—C14	1.456 (2)	C8A—C14A	1.490 (2)
C8—H8	0.9500	C8A—H8A	0.9500
C11—C16	1.392 (2)	C11A—C16A	1.393 (2)
C11—C12	1.4196 (19)	C11A—C12A	1.412 (2)
C12—C13	1.371 (2)	C12A—C13A	1.376 (2)
C13—C14	1.407 (2)	C13A—C14A	1.400 (2)
C13—H13	0.9500	C13A—H13A	0.9500
C14—C15	1.386 (2)	C14A—C15A	1.383 (2)
C15—C16	1.380 (2)	C15A—C16A	1.389 (2)
C15—H15	0.9500	C15A—H15A	0.9500
C16—H16	0.9500	C16A—H16A	0.9500
			
C11—O1—C1	116.64 (11)	C11A—O1A—C1A	117.43 (11)
C12—O2—C7	117.29 (11)	C12A—O2A—C7A	116.70 (11)
O1—C1—C2	109.40 (12)	O1A—C1A—C2A	108.22 (11)
O1—C1—H1A	109.8	O1A—C1A—H1A1	110.1
C2—C1—H1A	109.8	C2A—C1A—H1A1	110.1
O1—C1—H1B	109.8	O1A—C1A—H1A2	110.1
C2—C1—H1B	109.8	C2A—C1A—H1A2	110.1
H1A—C1—H1B	108.2	H1A1—C1A—H1A2	108.4
C1—C2—C3	109.01 (12)	C1A—C2A—C3A	110.77 (12)
C1—C2—H2A	109.9	C1A—C2A—H2A1	109.5
C3—C2—H2A	109.9	C3A—C2A—H2A1	109.5
C1—C2—H2B	109.9	C1A—C2A—H2A2	109.5
C3—C2—H2B	109.9	C3A—C2A—H2A2	109.5
H2A—C2—H2B	108.3	H2A1—C2A—H2A2	108.1
C4—C3—C2	114.72 (12)	C4A—C3A—C2A	113.41 (12)
C4—C3—H3A	108.6	C4A—C3A—H3A1	108.9
C2—C3—H3A	108.6	C2A—C3A—H3A1	108.9
C4—C3—H3B	108.6	C4A—C3A—H3A2	108.9
C2—C3—H3B	108.6	C2A—C3A—H3A2	108.9
H3A—C3—H3B	107.6	H3A1—C3A—H3A2	107.7
C5—C4—C3	112.93 (12)	C3A—C4A—C5A	113.56 (12)
C5—C4—H4A	109.0	C3A—C4A—H4A1	108.9
C3—C4—H4A	109.0	C5A—C4A—H4A1	108.9
C5—C4—H4B	109.0	C3A—C4A—H4A2	108.9
C3—C4—H4B	109.0	C5A—C4A—H4A2	108.9
H4A—C4—H4B	107.8	H4A1—C4A—H4A2	107.7
C4—C5—C6	112.80 (13)	C6A—C5A—C4A	112.45 (12)
C4—C5—H5A	109.0	C6A—C5A—H5A1	109.1
C6—C5—H5A	109.0	C4A—C5A—H5A1	109.1
C4—C5—H5B	109.0	C6A—C5A—H5A2	109.1
C6—C5—H5B	109.0	C4A—C5A—H5A2	109.1
H5A—C5—H5B	107.8	H5A1—C5A—H5A2	107.8
C5—C6—H6A	109.5	C5A—C6A—H6A1	109.5
C5—C6—H6B	109.5	C5A—C6A—H6A2	109.5
H6A—C6—H6B	109.5	H6A1—C6A—H6A2	109.5
C5—C6—H6C	109.5	C5A—C6A—H6A3	109.5
H6A—C6—H6C	109.5	H6A1—C6A—H6A3	109.5
H6B—C6—H6C	109.5	H6A2—C6A—H6A3	109.5
O2—C7—H7A	109.5	O2A—C7A—H7A1	109.5
O2—C7—H7B	109.5	O2A—C7A—H7A2	109.5
H7A—C7—H7B	109.5	H7A1—C7A—H7A2	109.5
O2—C7—H7C	109.5	O2A—C7A—H7A3	109.5
H7A—C7—H7C	109.5	H7A1—C7A—H7A3	109.5
H7B—C7—H7C	109.5	H7A2—C7A—H7A3	109.5
O3—C8—C14	125.69 (16)	O3A—C8A—C14A	125.5 (2)
O3—C8—H8	117.2	O3A—C8A—H8A	117.3
C14—C8—H8	117.2	C14A—C8A—H8A	117.3
O1—C11—C16	125.13 (13)	O1A—C11A—C16A	125.09 (13)
O1—C11—C12	115.47 (12)	O1A—C11A—C12A	115.56 (12)
C16—C11—C12	119.41 (14)	C16A—C11A—C12A	119.35 (13)
O2—C12—C13	125.44 (13)	O2A—C12A—C13A	124.85 (13)
O2—C12—C11	114.72 (12)	O2A—C12A—C11A	115.33 (12)
C13—C12—C11	119.84 (13)	C13A—C12A—C11A	119.82 (14)
C12—C13—C14	120.33 (13)	C12A—C13A—C14A	120.62 (14)
C12—C13—H13	119.8	C12A—C13A—H13A	119.7
C14—C13—H13	119.8	C14A—C13A—H13A	119.7
C15—C14—C13	119.53 (14)	C15A—C14A—C13A	119.56 (14)
C15—C14—C8	119.63 (14)	C15A—C14A—C8A	122.97 (15)
C13—C14—C8	120.84 (14)	C13A—C14A—C8A	117.47 (15)
C16—C15—C14	120.74 (14)	C14A—C15A—C16A	120.51 (15)
C16—C15—H15	119.6	C14A—C15A—H15A	119.7
C14—C15—H15	119.6	C16A—C15A—H15A	119.7
C15—C16—C11	120.14 (14)	C15A—C16A—C11A	120.14 (15)
C15—C16—H16	119.9	C15A—C16A—H16A	119.9
C11—C16—H16	119.9	C11A—C16A—H16A	119.9
			
C11—O1—C1—C2	−178.51 (12)	C11A—O1A—C1A—C2A	176.72 (12)
O1—C1—C2—C3	−179.57 (12)	O1A—C1A—C2A—C3A	176.23 (12)
C1—C2—C3—C4	176.08 (12)	C1A—C2A—C3A—C4A	177.66 (13)
C2—C3—C4—C5	−179.02 (12)	C2A—C3A—C4A—C5A	178.51 (13)
C3—C4—C5—C6	179.82 (13)	C3A—C4A—C5A—C6A	−179.53 (14)
C1—O1—C11—C16	−1.5 (2)	C1A—O1A—C11A—C16A	−3.7 (2)
C1—O1—C11—C12	178.79 (12)	C1A—O1A—C11A—C12A	176.50 (12)
C7—O2—C12—C13	−0.3 (2)	C7A—O2A—C12A—C13A	7.2 (2)
C7—O2—C12—C11	179.93 (12)	C7A—O2A—C12A—C11A	−173.04 (12)
O1—C11—C12—O2	0.82 (18)	O1A—C11A—C12A—O2A	−0.93 (18)
C16—C11—C12—O2	−178.89 (13)	C16A—C11A—C12A—O2A	179.30 (13)
O1—C11—C12—C13	−179.01 (12)	O1A—C11A—C12A—C13A	178.89 (12)
C16—C11—C12—C13	1.3 (2)	C16A—C11A—C12A—C13A	−0.9 (2)
O2—C12—C13—C14	179.44 (14)	O2A—C12A—C13A—C14A	−179.87 (13)
C11—C12—C13—C14	−0.8 (2)	C11A—C12A—C13A—C14A	0.3 (2)
C12—C13—C14—C15	−0.2 (2)	C12A—C13A—C14A—C15A	0.4 (2)
C12—C13—C14—C8	179.23 (14)	C12A—C13A—C14A—C8A	179.73 (13)
O3—C8—C14—C15	−179.18 (18)	O3A—C8A—C14A—C15A	4.4 (3)
O3—C8—C14—C13	1.4 (3)	O3A—C8A—C14A—C13A	−174.92 (18)
C13—C14—C15—C16	0.6 (2)	C13A—C14A—C15A—C16A	−0.6 (2)
C8—C14—C15—C16	−178.82 (15)	C8A—C14A—C15A—C16A	−179.89 (15)
C14—C15—C16—C11	−0.1 (2)	C14A—C15A—C16A—C11A	0.1 (2)
O1—C11—C16—C15	179.45 (14)	O1A—C11A—C16A—C15A	−179.05 (14)
C12—C11—C16—C15	−0.9 (2)	C12A—C11A—C16A—C15A	0.7 (2)
